# tRF3008A suppresses the progression and metastasis of colorectal cancer by destabilizing FOXK1 in an AGO-dependent manner

**DOI:** 10.1186/s13046-021-02190-4

**Published:** 2022-01-22

**Authors:** Ying Han, Yinghui Peng, Shanshan Liu, Xinwen Wang, Changjing Cai, Cao Guo, Yihong Chen, Le Gao, Qiaoqiao Huang, Min He, Edward Shen, Jie Long, Jian Yu, Hong Shen, Shan Zeng

**Affiliations:** 1 Department of Oncology, Xiangya Hospital, Central South University, Changsha, 410008 Hunan China; 2 National Clinical Research Center for Geriatric Disorders, Xiangya Hospital, Central South University, Changsha, 410008 Hunan China; 3 Department of Pathology, University of Pittsburgh School of Medicine, 5117 Centre Ave, Pittsburgh, PA 15213 USA; 4 Key Laboratory for Molecular Radiation Oncology of Hunan Province, Xiangya Hospital, Central South University, Changsha, 410008 Hunan China; 5 Department of Life Science, McMaster University, Hamilton, ON L8S 4L8 Canada

**Keywords:** tRNA-derived fragments (tRFs), tRF3008A, FOXK1, AGO, Colorectal cancer

## Abstract

**Background:**

tRNA-derived fragments (tRFs) have been shown to have critical regulatory roles in cancer biology. However, the contributions of tRFs to colorectal cancer (CRC) remain largely unknown.

**Methods:**

tRF3008A (a tRFRNA derived from tRNA^Val^) was identified by RNA sequencing and validated by quantitative reverse transcription PCR. The role of tRF3008A in CRC progression was assessed both in vitro and in vivo, and its downstream target genes were identified and validated in CRC cells. RNA pull-down with mass spectrometry and AGO-RIP were used to confirm the interaction of tRF3008A and AGO proteins. The clinical implications of tRF3008A were assessed in CRC tissues and blood samples.

**Results:**

The expression of tRF3008A was reduced in colorectal cancer, and its reduction was significantly correlated with advanced and metastatic disease in CRC. Patients with low tRF3008A expression showed significantly shorter DFS, and multivariate analysis identified tRF3008A as an independent prognostic biomarker in CRC. Functionally, tRF3008A inhibits the proliferation and migration of CRC in vivo and in vitro by repressing endogenous FOXK1, a positive regulator of the Wnt/β-catenin pathway. Mechanistically, tRF3008A binds to AGO proteins as a guide to destabilize oncogenic *FOXK1* transcript.

**Conclusions:**

tRF3008A suppresses the metastasis and progression of colorectal cancer by destabilizing FOXK1 in an AGO-dependent manner.

**Supplementary Information:**

The online version contains supplementary material available at 10.1186/s13046-021-02190-4.

## Background

Colorectal cancer (CRC) is one of the most lethal diseases and the third leading cause of cancer-related death [1]. Approximately 20 to 30% of human colorectal cancer cases present with metastasis at the time of diagnosis and more than half of CRC patients will develop metastasis mostly to the liver and lung. In recent years, the CRC patient population is rapidly shifting younger, which becomes a new challenge [2]. CRC is a disease with strong environmental associations as well as genetic risk factors. Signatures including genomic instability and oncogene mutations are already in development for clinical biomarkers in colorectal cancer [3]. Although the molecular mechanisms under aberrant biological processes are not yet fully understood, continued efforts to investigate novel diagnostic markers and therapeutic targets will lead to the translation of these insights into the clinical arena.

Noncoding RNAs have emerged as important regulators in diverse aspects of biology. Transfer RNAs (tRNAs) are a type of most extensively modified and structured RNA that have a well-defined role in mRNA translation via linking codons on messenger RNAs to their corresponding amino acids in the ribosome [4]. tRNAs have a strong secondary and tertiary structure, which can be further processed into tRNA-derived fragments (tRFs). Notably, tRNA and tRFs abundance is cell- specific and tissue-specific in different kinds of disease and the functions have been implicated in a wide range of biological processes [5]. tRFs generated by precise splicing of tRNAs and pre-tRNAs are present in most organisms. They belong to a family of short noncoding RNAs (ncRNAs) with a length about 14–30 nts. Based on the position of the tRNA cleavage site, tRFs can be classified into three main types: 5-tRFs, 3-tRFs and 1-tRFs. The 5-tRF series is derived from the 5′- ends of mature tRNAs by a cleavage of the D loop and the tRF-3 series is formed by specific cleavage of the 3′-ends of mature tRNAs in the T loop, while 1-tRF series is resulted from cleavage of the 3′-trailer fragment of pre-tRNAs [6]. The function of tRFs in regulating translation was documented in early studies by Paul Anderson’s group [5]. Recent emerging studies have further expanded our understanding of distinct features of tRFs ranging from tumorigenesis, stem cell biology and stress response [7–10].

Argonaute (AGO) proteins are members of a highly conserved protein family that can be found in all domains of life [11]. Argonaute proteins are key factors of multiple transcriptional and posttranscriptional gene regulatory pathways by forming ribonucleoprotein complexes (RNPs) with small RNAs [12]. Some tRF-3 s and tRF-5 s could bind to AGO proteins in a manner similar to that of miRNAs [13], suggesting a potential function of this tRF–Argonaute protein interaction to regulate gene expression.

In this study, we identified a novel 3′- tRNA^val-^derived tRF3008A as a critical regulator of cancer growth and progression in colorectal cancer cells. By combining molecular, biochemical, and computational approaches, we determined that tRF3008A suppressed Wnt/β-catenin signaling by directly interacting with FOXK1. Moreover, we found that tRF3008A binds to AGO, resulting in repressed expression of FOXK1, thereby suppressing progression of colorectal cancer cells.

## Methods

### Clinical samples

Human specimen collection and usage was approved by the Ethics Committee of Xiangya Hospital. In total, 70 pairs of CRC and corresponding adjacent non-tumorous tissues (ANT) were obtained via surgical resection from patients who did not undergo preoperative treatment. Informed consent was obtained from each patient according to the policies of the committee. Frozen samples were used in RNA-seq and RT-PCR. Among them, three pairs (cohort 1) were used for RNA-seq, and 45 pairs (cohort 2) were used for tRFs validation. The detailed clinicopathological features were described (Table 1).

**Table 1:** Correlation between tRF3008A expression and the clinical pathological characteristics in CRC

Clinical pathological indexes		CRC tissues (*n* = 45)		*P*	Blood samples (*n* = 34)		
		tRF3008A(High)	tRF3008A (low)		tRF3008A(High)	tRF3008A (low)	*P*
Age	≤51	13	11	0.449	8	7	0.535
	> 51	9	12		9	12	
Gender	Male	10	7	0.299	12	9	0.290
	Female	12	16		5	8	
Tumor size	≤5 cm	11	5	0.048	10	3	0.014
	> 5 cm	11	18		7	14	
Tumor differentiation	Well	15	7	0.011	7	8	0.730
	Poor	7	16		10	9	
Tumor invasion	T1 + T2	12	14	0.668	4	5	0.698
	T3 + T4	10	9		13	12	
Tumor location	Distal	17	18	0.937	13	11	0.452
	Proximal	5	5		4	6	
ECOG score	0–1	20	18	0.242	12	13	0.698
	≥2	2	5		5	4	

Blood samples were obtained from 34 CRC patients. Informed consent was received from those patients. All colorectal cancer diagnoses were verified via histopathological analysis of the tumors, and the tumor staging was based on the tumor-node-metastasis (TNM) system (Gabriel, Stephen, & Armando, 2018). The basic clinical characteristics of the subjects are listed in Table 1. Whole-blood samples (10 ml) were collected before and after surgery into purple-top tubes (SANLI, China, 20152220045). Plasma was prepared within 1 h of collection by centrifuging at 1300×g for 10 min, at 4 °C, and stored at − 80 °C for further experiments. The entire sample processing procedure was performed on ice. This study was approved by the Ethics Committee of the Xiangya Hospital of Central South University.

### tRFs pretreatment and cDNA synthesis

The rtStar™ tRF&tiRNA Pretreatment Kit (Cat# AS-FS-005, Arraystar) was used to remove the modifications that interfere with small RNA-seq library construction or qPCR. The kit provides the reagents necessary to perform the following treatments before library preparation for the total RNA samples: 3′-aminoacyl (charged) deacylation to 3′-OH for 3′ adaptor ligation; 3′-cP (2′,3′-cyclic phosphate) removal to 3′-OH for 3′ adaptor ligation; 5′-OH (hydroxyl group) phosphorylation to 5′-P for 5′-adaptor ligation; and m1A and m3C demethylation for efficient reverse transcription. The rtStar™ First-Strand cDNA Synthesis Kit (5′ and 3′ Adaptors) (Cat# AS-FS-003-02, Arraystar) was used to create cDNA libraries from small RNAs for qPCR detection according to the manufacturer’s instructions. The kit provides the reagents to sequentially ligate 3′-Adaptor with its.

5′-end to the 3′-end of the RNAs, and 5′-Adaptor with its 3′-end to the 5′-end of the RNAs. The non-ligation ends of 3′ and 5’Adaptors are blocked by modification. 3′ Adaptor contains a universal priming site for Reverse Transcription (RT) Primer.

### Real-time PCR for tRFs

qRT-PCR was performed using the 2X PCR master mix(Arraystar). The reactions were performed with a 7500 Fast Real-Time PCR System. The average expression levels of tissue and plasma tRFs were normalized against U6, the levels of tRFs were calculated using 2^−ΔCt^ or 2^−ΔΔCt^ method for relative quantification of expression, in which ΔCt = Ct(tRFs)–Ct(U6) and ΔΔCt = ΔCt(case) -ΔCt(control). The list of primers is described in the Supplementary Materials and Methods.

### Cell culture

The human colon cell line FHC and the human colon cancer cell lines HCT116, RKO, SW48, HT29, LOVO, LS174T and SW480 were obtained from the Institutes of Biomedical Sciences (IBS, Shanghai, China). Cells were cultured in DMEM (Gibco, Grand Island, NY) or RPMI 1640 (HyClone, Logan, UT) supplemented with 10% fetal bovine serum (FBS).

### Exogenous tRF and antisense LNA transfection

tRF mimetics (Robbio, Guangzhou, China) were synthesized and tRF antisense LNA oligonucleotides were constructed by Exiqon. tRF antisense LNA oligonucleotides or synthetic tRF mimetic at a final concentration of 50 nM were transfected using Lipofectamine 3000 in Reduced Serum Media (Life Technologies). The transfection medium was replaced with fresh medium 6 h post transfection. After incubated for 48 h, cells were subjected to in vitro and in vivo studies.

### Cell proliferation, apoptosis (flow cytometry), migration and invasion assays

CCK8, Edu, flow cytometry, invasion, migration and apoptosis assays were performed in CRC cell lines according to the manufacturer’s instructions, as described in the Supplementary Materials and Methods.

### Western blotting

The procedures and the primary antibodies used are detailed in the Supplementary Materials and Methods.

### Immunofluorescence (IF) staining and immunohistochemistry (IHC)

IF staining and IHC were performed and scored according to the percentage of positively stained cells and staining intensity as described in the Supplementary Materials and Methods.

### In vivo tumorigenicity and metastasis studies

All mice were treated humanely, and the protocols for treating animals were approved by the Medical Experimental Animal Care Commission of Central South University. HCT116 cells transfected with tRF3008A mimetics and HT29 cells transfected with tRF3008A-LNA were subcutaneously injected into the right flank regions of female BALB/c nu/nu mice (6 mice/group, 4 weeks of age, 5× 10^6^ per mouse). Tumor growth was monitored every 3 day after inoculation according to the following formula: V (mm^3^) = (L × W^2^) × 0.5 (L: tumor length, W: width), and all mice were sacrificed 3 weeks after injection. The final tumors were removed and calculated. IHC staining was performed with markers of cell proliferation, apoptosis and invasion (ki-67, Cleaved Caspase-3 and MMP9, respectively). According to the manufacturer’s protocol, a TUNEL assay kit (Roche, Basel, Switzerland) was used to detect cell apoptosis. Fluorescence images were obtained using a Carl Zeiss confocal microscope (Weimar, Germany). For metastatic colonization models, HCT116 cells transfected with tRF3008A mimetics or scrambled tRF mimetic control in 100 ml PBS were injected via tail-vein into female nude mice (10 mice/group, 4 weeks of age, 1 × 10^6^ per mouse) and metastasis was recorded and imaged 4 weeks after injection. All animal studies were conducted according to a protocol approved by the Institutional Animal Care and Use Committee (IACUC) at Central South University.

### Dual-luciferase reporter assay

Luciferase assays were performed in 96-well plates. The cells were plated at 70% confluence. Luciferase reporter gene plasmid and overexpressed plasmid were transfected 24 h later. The diluted DNA and tRFs plasmids were mixed with the transfection reagent, respectively, and incubated for 20 min at room temperature and then added to each cell samples. After 48 h of plasmid co-transfection. The luciferase signals were measured using 50 μL of the Stop&Glo Reagent following the instructions provided by Obio Technology. The Renilla luciferase levels were normalized to firefly luciferase levels.

### RNA immunoprecipitation (RIP) and RNA pull-down

For Argonaute immunoprecipitation from tRF overexpressing cells or tRF knock down cells were performed as described in the Magna RIP™ RNA-Binding Protein Immunoprecipitation Kit (Millipore 17–700). 5 × 10^7^ cells were incubated with anti–pan-AGO antibody (MABE56; Millipore) or control IgG overnight with rotation at 4 °C. After immunoprecipitation of RNA-binding Protein-RNA complexes, RNA was extracted by phenol chloroform and further subjected to qRT-PCR analysis.

RNA pull-down assays were performed according to the instructions by Pierce™ Magnetic RNA-Protein Pull-Down Kit (Thermo Scientific™,20164). 1 × 10^7^ cells were washed in ice-cold phosphate-buffered saline and cell lysates were prepared using standard lysis buffers and incubated with 3 μg of biotinylated RNA oligo probes against endogenous or ectopically expressed tRF3008A at room temperature for 2 h. And then the RNA-Binding Protein Complexes were wash and the bound proteins in the complexes were further subjected to Western blotting or LC-MS/MS analysis.

### Statistical analysis

Statistical analysis was performed with GraphPad Prism 7.0 software. All data are presented as the means ± SD, and two-tailed unpaired or paired Student’s t test and ANOVA (Dunnett’s or LSD post hoc test) was used accordingly. Spearman correlation analysis was used to determine the relationship between the indicated mRNAs and tRFs. Survival curves were calculated using the Kaplan-Meier method and the log-rank test. The Cox proportional hazards regression model was used to identify predictive factors that independently affected the prognosis of colon cancer. All tests were two-sided, and *p* < 0.05 was considered statistically significant.

## Results

### Systematic identification of Cancer-related tRFs in CRC

**Fig. 1:**
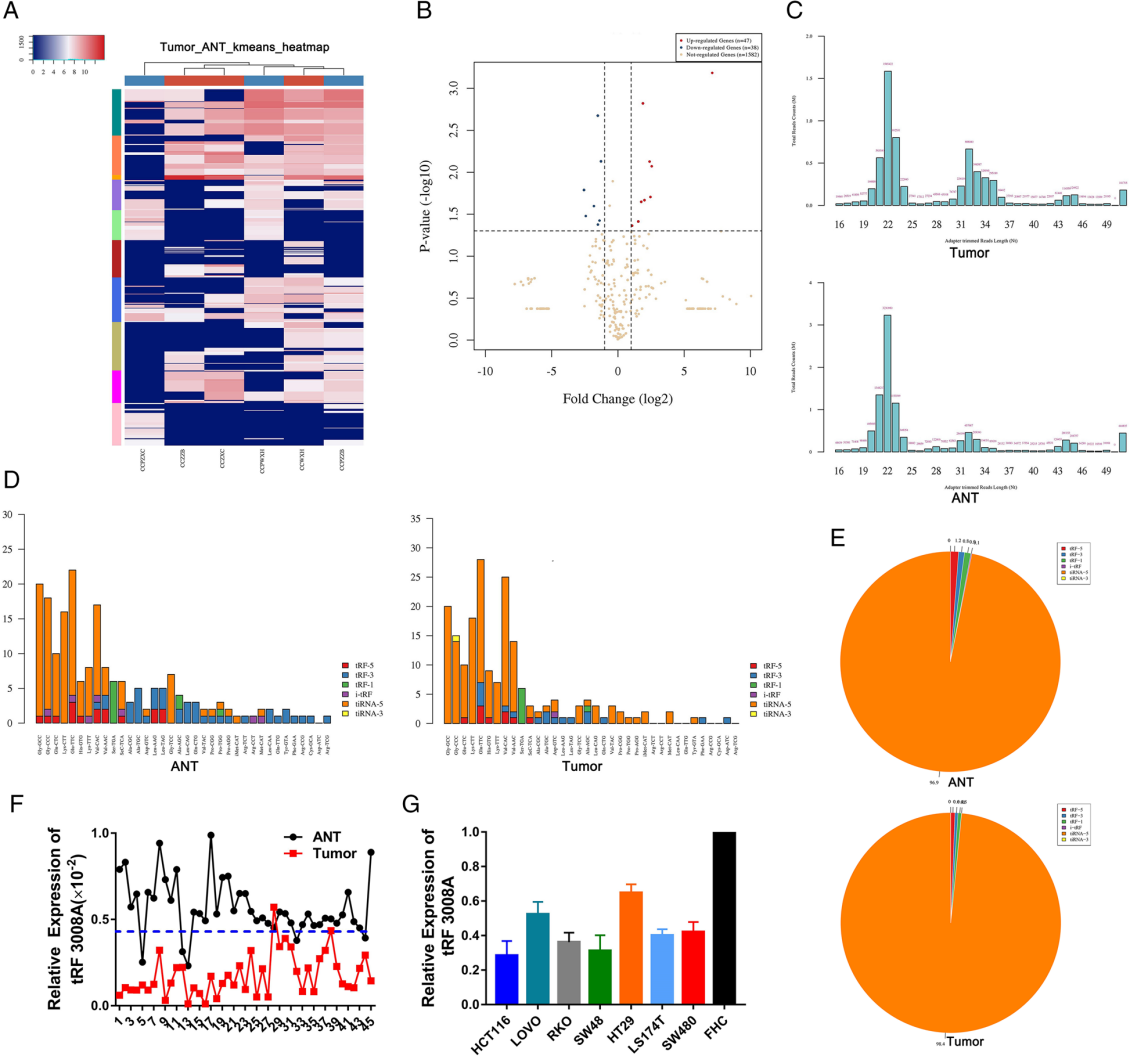
Genome-wide profiling of tRFs in colorectal cancer. **A&B** Clustered heat map and volcano plots of the differentially expressed tRFs in three pairs of CRCs (CCWXH, CCZZB, CCZXC) and adjacent non-tumorous tissues (CCPWXH, CCPZZB, CCPZXC). Rows represent tRNAs; while columns represent tissues (top red: tumors; top blue: ANTs). **C** The length distribution of tRFs in CRC and adjacent non-tumorous tissues. **D&E** Genomic origin of the tRFs identified in human CRC and adjacent non-tumorous tissues. **F**. RT-qPCR to validate tRF3008A expression in 45 pairs of CRCs and adjacent non-tumorous tissues. **G**. RT-qPCR to validate tRF3008A expression in 7 CRC cell lines and human colon epithelial cell line. Statistical significance is measured using Student’s t test

We first characterized tRNA-derived fragments in human colorectal cancer using tRF & tiRNA-seq analysis from three pairs of human CRCs and adjacent non-tumorous tissues. Sequencing was carried out, and expression levels were reported according to log_2_FC of significantly differentially expressed RNAs. We detected 1667 distinct tRFs in total and 16 differentially expressed tRFs and tiRNAs, among which 9 tRFs from 47 tRNAs were upregulated and 7 tRFs from 38 tRNAs were downregulated in CRCs compared with ANT (Table S1). They were displayed by a hierarchical clustering heatmap and a Volcano plot (Fig. 1A & B). Venn diagram shows the commonly and specifically expressed genes. The commonly expressed genes represent the TPM values which were not zero in both two groups, and the specifically expressed genes represent the TPM values which were zero in one group while not zero in another group (Fig. S1A). After passing the Solexa CHASTITY filtering and 3′-adapter filter, the 3′-adapter trimmed reads were recorded for each unique read between CRC tissues and adjacent non-tumorous tissues. A bar graph is used to show the sequence read length distribution (Fig. 1C). To further investigate the distinct distribution of tRFs between CRC tissues and adjacent non-tumorous tissues, tRF-5 showed the highest expression in both CRC tissues and ANT, and the expression of tRF-3 was higher in adjacent non-tumorous tissues than those in CRC tissues (Fig. 1D & E).

To verify the RNA-seq results, the top 3 tRFs(tRF3008A, tRF1001 and tRF3001) with the greatest difference were selected as candidate tRFs after excluding the very low abundance tRFs. RT-qPCR using tRF-specific divergent primers was performed to examine the expression of 3 top tRFs in 10 pairs of CRC and adjacent non-tumorous tissues. The results showed that tRF3008A(AS-tDR-000076) was of the most pronounced difference in expression, which was consistent with the RNA-seq results (Fig. S1B). This finding was confirmed by RT-qPCR in 45 pairs of CRCs tissues (Fig. 1F).

### tRF3008A suppresses colorectal cancer cell growth in vitro and in vivo

The expression of tRF3008A in FHC cell line (Fetal colon cell line) and 7 human CRC cell lines was examined by RT-qPCR. Among these 8 cells lines, the expression of tRF3008A was highest in the FHC cells, and for the CRC cells, HCT116 cells exhibited the lowest expression of tRF3008A while HT29 cells showed the highest expression (Fig. 1G). Northern blot was performed to detect the expression of mature form of tRNA^val^ and tRF3008A, showing that the expression of tRF3008A was independent of tRNA^val^ levels (Fig. S1C). For functional studies, HCT116 cells and HT29 cells were selected.

**Fig. 2:**
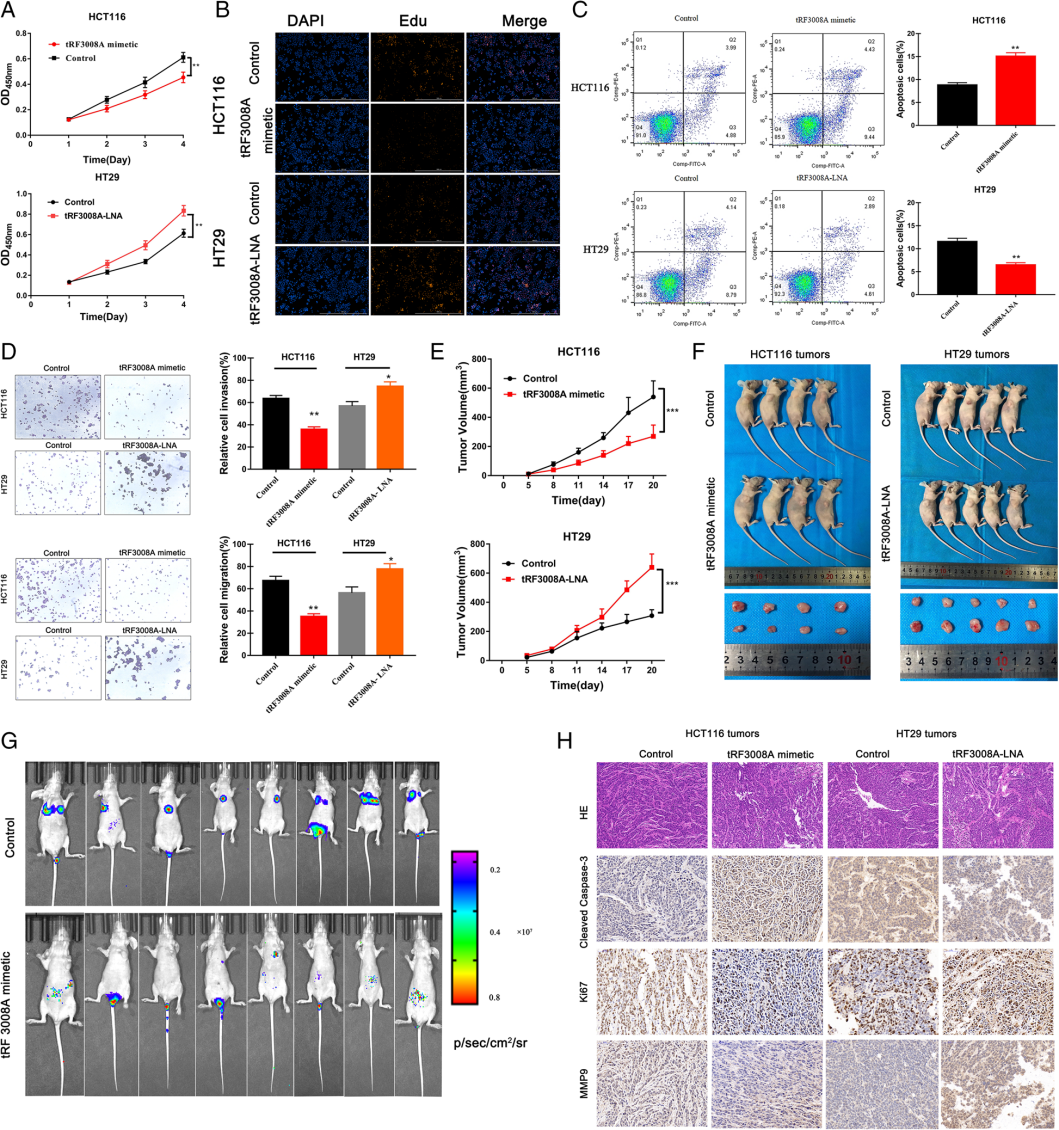
tRF3008A inhibits colorectal cancer growth and migration in vitro: HCT116 cells were transfected with scrambled tRF mimetic control or tRF3008A mimetic, and HT29 cells were transfected with scrambled LNA control or tRF3008A-LNA. **A&B** Cell Counting Kit-8 and EdU assays were performed to evaluate cell proliferation. **C** Flow cytometry analysis of the apoptosis of HCT116 and HT29 cells with different expression levels of tRF3008A. **D** Cell migration and invasion abilities were evaluated by transwell migration and Matrigel invasion assays, respectively. **E&F** HCT116 cells or HT29 cells with different tRF3008A expression levels were subcutaneously implanted into nude mice, and then tumor growth was examined by measuring the tumor volume (every 3 days; *n* = 5). Line graph showing the tumor volume (mm^3^) from day 0 to day 21 after injection. Error bars represent the SD from 5 mice. For statistical analysis, Student’s t-test (two-sided, paired) was used. Three weeks after subcutaneous inoculation, the average tumor volumes in each group were calculated using the following formula: V (mm^3^) = (L × W^2^) × 0.5 (L: tumor length, W: width). **G** Four weeks after tail vein injection with HCT116 cells or HT29 cells with different tRF3008A expression levels, the luciferase signal intensities of mice were measured to identify metastasis foci (*n* = 10). **H H&E** staining for tissue morphology and immunohistochemistry staining for markers of proliferation (Ki67), apoptosis (CC3, cleaved caspase-3), and invasion (MMP9) in subcutaneous tumors. Data are represented as the means ± S.D. of at least three independent experiments. Statistical significance is measured using Student’s t test. Scare bar = 1000 μm. *: *P* < 0.05, **: *P* < 0.01, ***: *P* < 0.001

We succeeded in overexpressing tRF3008A in HCT116 cells via tRF3008A mimetic transfection and knocking down tRF3008A in HT29 cells by using tRF3008A-LNA. Northern blot proved that the transfection of tRF3008A mimetic or tRF3008A-LNA didn’t change the mature tRNA^Val^ levels (Fig. S1D). Cell proliferation was measured with CCK8 and EdU assays, and the results revealed a significant reduction in the growth by the tRF3008A mimetic compared with the negative control and an increase with tRF3008A knock down (Fig. 2A & B, Fig.S2A). The active caspase-3 staining assay showed higher levels in the HCT116 tRF3008A mimetic group and HT29 control group (Fig.S2B). In addition, more apoptotic cells were present in the relatively high tRF3008A group than in the relatively low tRF3008A group in the HCT116 and HT29 cell lines, respectively (Fig. 2C). Moreover, transwell assays without or with Matrigel demonstrated that tRF3008A silencing markedly improved HT29 cell migration and invasion while tRF3008A mimetic impeded HCT116 cell migration and invasion (Fig. 2D). These data collectively indicate that overexpressing tRF3008A can delay the progression of CRC cells.

To explore the effects of tRF3008A in vivo, HCT116 cells transfected with tRF3008A mimetic and HT29 cells transfected with tRF3008A-LNA and respective negative control cells were injected into the right flanks of nude mice (5 mice in each group). qPCR further confirmed the expression of tRF3008A in subcutaneous tumors (Fig. S2C). The results showed that the growth of tumors from the higher tRF3008A groups was significantly inhibited (Fig. 2E & F). Three weeks later, a TUNEL assay was performed on subcutaneous tumors, which indicated more apoptotic CRC cells in subcutaneous tumors from the HCT116 tRF3008A mimetic group and HT29 control group than in their respective counterparts (Fig.S2D & E).

Subsequently, metastatic models were established by intravenous tail injection of those cells into nude mice. As the cells expressed firefly luciferase, in vivo imaging system (IVIS) was used to dynamically monitor the metastases. Metastases were observed in the HCT116 control group, but the HCT116 tRF3008A mimetic group had significantly fewer metastases (Fig. 2G).

IHC staining using antibodies for specific detection of Ki67 (proliferation), cleaved caspase-3 (apoptosis) and MMP-9 (invasion) further confirmed the inhibitory effect of tRF3008A on tumor progression (Fig. 2H).

### tRF3008A suppresses Wnt/β-catenin signaling by targeting FOXK1

**Fig. 3:**
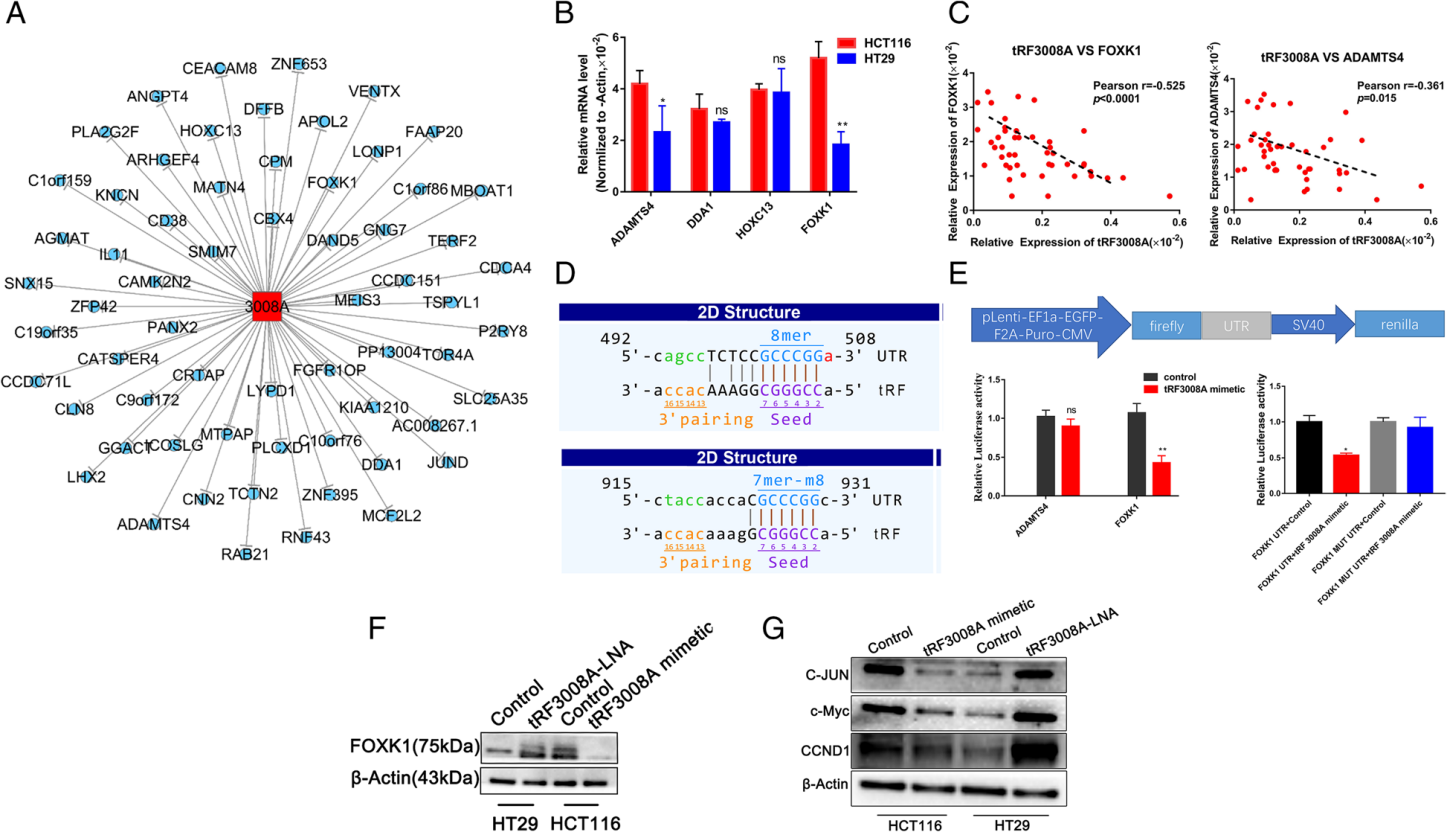
tRF3008A regulates FOXK1 expression. **A** Potential target genes of tRF3008A as predicted by TargetScan, miRanda, and TargetRank. **B** RT-qPCR analysis of 4 potential target genes in HCT116 cells and HT29 cells. **C** qPCR was performed to evaluate the expression correlation between tRF3008A and its potential targets (ADAMTS4 and FOXK1) in CRC tissues. (*n* = 45); Spearman’s rank correlation test was used. **D** Sequences of tRF3008A and its potential binding site at the 3′UTR of ADAMTS4 mRNA (bottom) and FOXK1 mRNA (top). **E** Dual-luciferase assays of candidate target genes of tRF3008A (left); dual-luciferase assays showing tRF3008A-mediated repression of the wild-type UTR (FOXK1-UTR) or mutant UTR (FOXK1-UTR-mut) (right). **F** Western blot analysis of FOXK1 in cells overexpressing tRF3008A (HCT116) or cells transfected with tRF3008A-LNA (HT29) and in their corresponding control cells. **G** Western blot analysis of downstream Wnt genes (C-JUN, c-Myc and CCND1) in control cells and cells expressing tRF3008A (HCT116) or cells transfected with tRF3008A-LNA (HT29). *: *P* < 0.05, **: *P* < 0.01

To select and identify the downstream targets of tRF3008A, bioinformatics analysis was performed using mRNA target-predicting algorithms (Fig. 3A). The related genes that showed the greatest overlap among these algorithms were four genes (ADAMTS4, DDA1, HOXC13, and FOXK1) and selected for further analysis. Next, we detected the expression levels of these genes in HCT116 cells and HT29 cells. ADAMTS4 and FOXK1 showed the most strikingly differential expression in tRF3008A-Low HCT116 cells and tRF3008A-High HT29 cells (Fig. 3B). We further performed qPCR to investigate the expression correlation of tRF3008A and these four genes in CRC. The results indicated that FOXK1 had negative correlation with tRF3008A expression in CRC while the correlation between ADAMTS4 and tRF3008A was poor although significant (Fig. 3C), but the expression of DDA1 and HOXC13 showed no significant correlation with tRF3008A expression (Fig. S3A & B). To confirm a direct relationship between tRF3008A and the predicted target genes, we cloned the 3′-UTR of these potential genes into a dual-luciferase vector (Fig. 3D&E). Firstly, we performed a luciferase assay by co-transfecting tRF3008A mimics with the Luc-FOXK1 or Luc-ADAMTS4 in HEK-293 T cells. Compared with the control group, tRF3008A mimics reduced the Luc-FOXK1 luciferase reporter activity but not the luciferase reporter activity of Luc-ADAMTS4 (Fig. 3E, Left). This experiment demonstrates the role of tRF3008A in regulation of FOXK1 through 3’UTR but not ADAMTS4. We next generated mutations in the binding site to abrogate the tRF3008A-FOXK1 3′UTR interaction, we found that tRF3008A selectively suppressed the reporter driven by WT-3′-UTR of FOXK1 (Fig. 3E, Right). We further confirmed that tRF3008A mimetic treatment decreases protein levels of FOXK1 in HCT116 cells, while tRF3008-LNA treatment increases levels of FOXK1 in HT29 cells. (Fig. 3F). According to previous reports, FOXK1 positively regulates Wnt/β-catenin signaling [14]. We hypothesized that tRF3008A inhibits Wnt/β-catenin signaling through FOXK1. Activation of the Wnt/β-catenin signaling pathway drives colorectal cancer growth by deregulating the expression of many downstream targets, such as c-JUN, c-Myc, cyclin D1, CD44 and Axin2. We selected three well-established targets (C-JUN, c-Myc, cyclin D1) and found their expression was strikingly downregulated in HCT116 cells upon tRF3008A mimetic treatment, and upregulated in HT29 cells upon tRF3008A knock down (Fig. 3G).

**Fig. 4:**
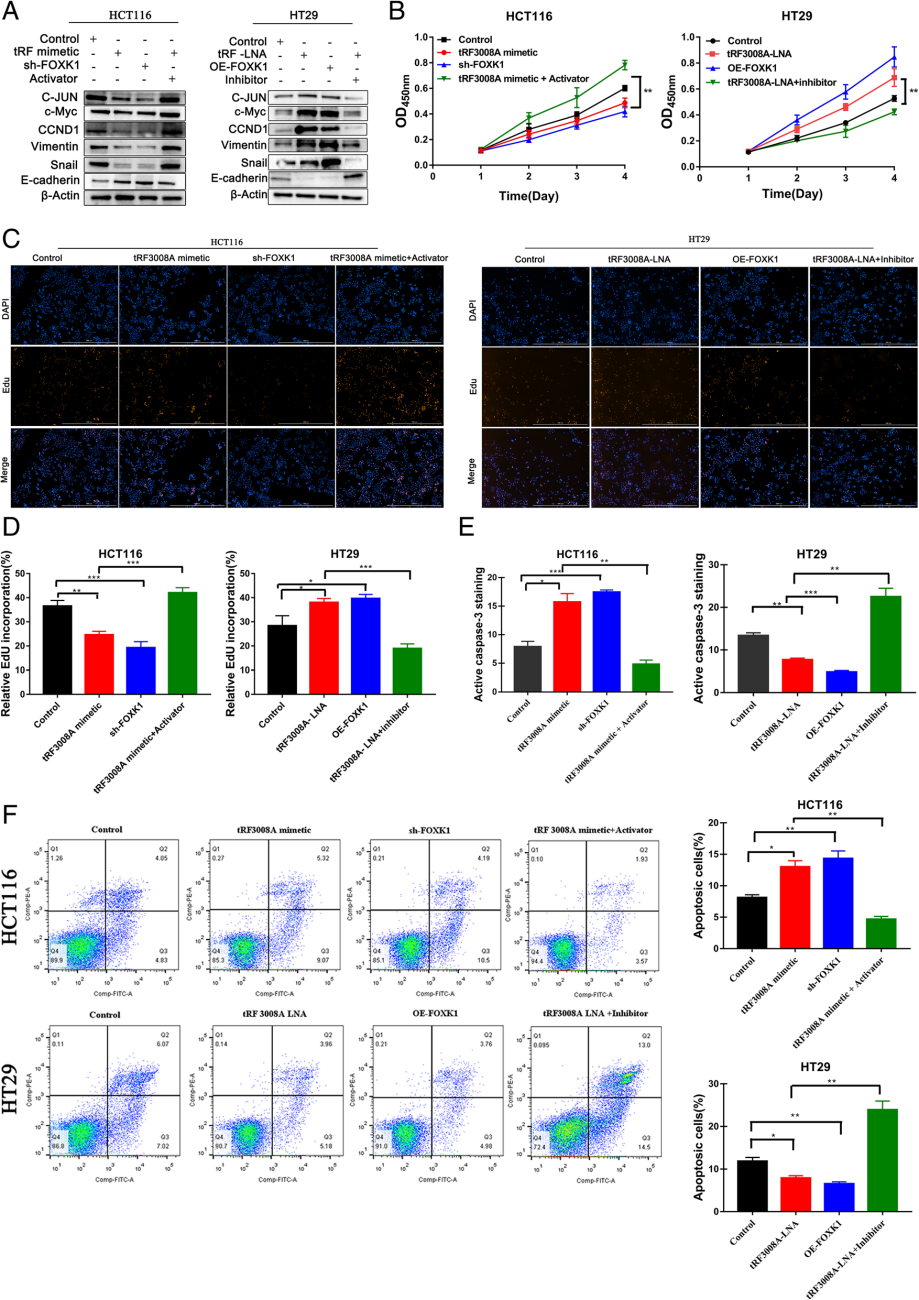
tRF3008A inhibits the growth and EMT of CRC through the FOXK1/Wnt pathway. HCT116 cells were co-transfected with tRF3008A mimetic and sh-FOXK1 in the presence or absence of a Wnt pathway activator (Wnt agonist 1); HT29 cells were co-transfected with tRF3008A-LNA and OE-FOXK1 in the presence or absence of a Wnt pathway inhibitor (IWR-1-endo). **A** Western blot analysis of the levels of proteins related to the Wnt/β-catenin signaling pathway (C-JUN, c-Myc and CCND1) and EMT (Vimentin, snail and E-cadherin). β-Actin was used as a loading control. **B** Proliferation assessed using the Cell Counting Kit-8 assay. **C&D** EdU assays were performed to evaluate cell proliferative ability. **E** Active caspase-3 was analyzed by immunostaining (red). **F** Apoptosis was evaluated by flow cytometry analysis. Data are presented as the means ± S.D. Statistical significance is measured using Student’s t test. *: *P* < 0.05, **: *P* < 0.01, ***: *P* < 0.001

To test the role of the tRF3008A/FOXK1 axis on Wnt signaling, we transfected HCT116 cells with sh-FOXK1 and found it significantly suppresses Wnt signaling as the tRF3008A mimetic treatment, which was diminished by the Wnt agonist 1(S8178) (Fig. 4A & S4A, left). Conversely, overexpression FOXK1 (OE-FOXK1) in HT29 cells induced Wnt signaling similar to the tRF3008A-LNA treatment, which was ameliorated by the Wnt pathway inhibitor IWR-1-endo (S7086) (Fig. 4A & S4A, Right). The growth of HCT116 cells was inhibited by sh-FOXK1 with increased apoptosis, similar to tRF3008A mimetic treatment, which was reduced by the Wnt activator treatment (Fig. 4B-F, Fig. S4B). The growth of HT29 cells was enhanced by OE-FOXK1 with reduced apoptosis, similar to tRF3008A-LNA, which was reduced by the Wnt inhibitor treatment (Fig. 4B-F, Fig. S4B). Transwell assays showed that the Wnt inhibitor effectively reverses the invasion and migration of CRC cells with tRF3008A silencing (Fig. S4C & D). Collectively, these observations demonstrate that tRF3008A inhibits the growth of CRC, at least partly, through the FOXK1/WNT pathway.

### tRF3008A destabilizes FOXK1 in an AGO-dependent manner

**Fig. 5:**
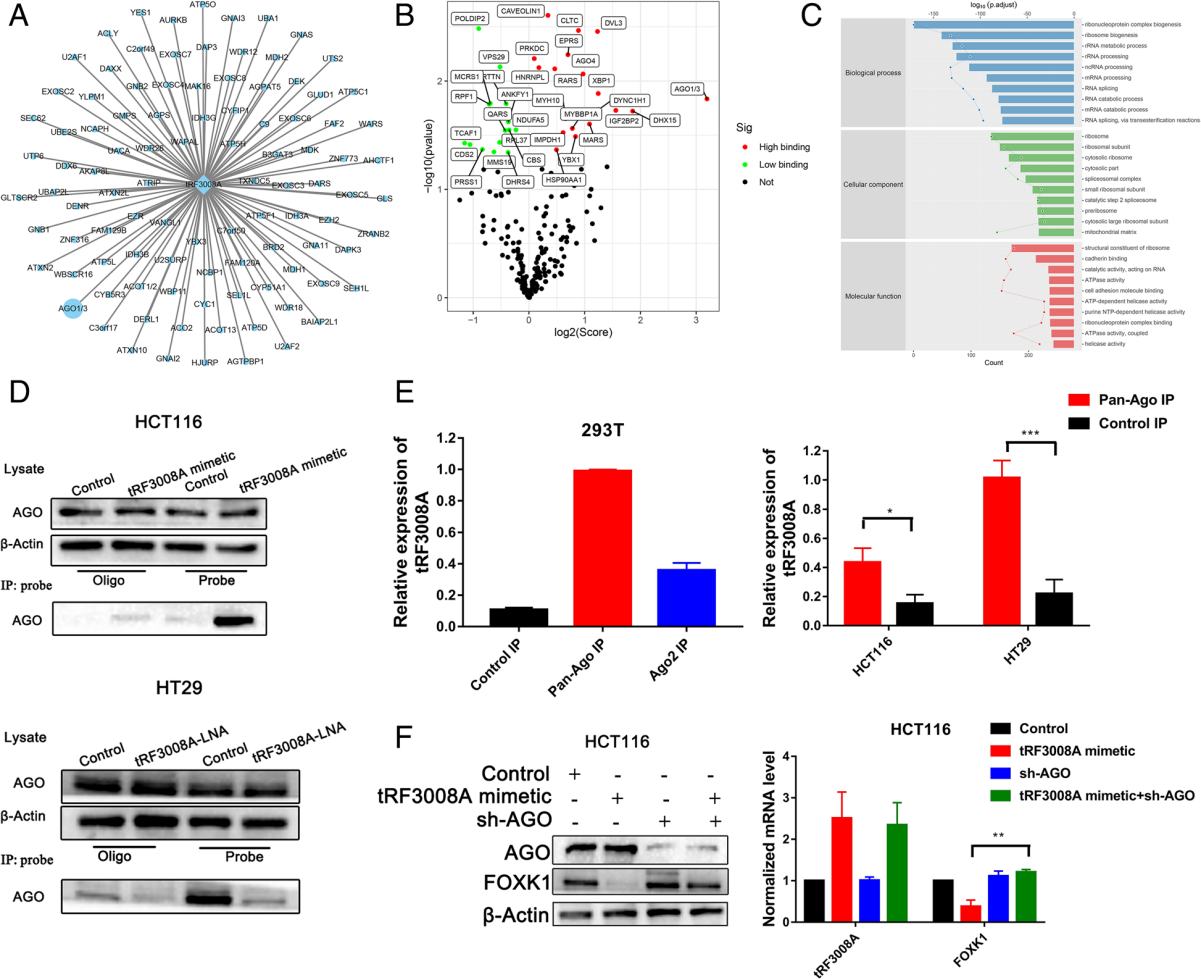
tRF3008A modulates FOXK1 in an AGO-dependent manner. tRF3008A affinity purification-mass spectrometry assay was performed as follows: **A** Overview of the tRF3008A-protein interactome. **B** Significant protein enrichment summary with a volcano plot. **C** GO enrichment analysis was performed to show protein enrichment classification information. **D** Protein lysates prepared from control and tRF3008A mimetic-transfected HCT116 cells were subjected to Western blot with antibody against AGO and to pull-down of AGO with the tRF3008A probe and oligo (Top); Protein lysates prepared from control and tRF3008A-LNA-transfected HT29 cells were subjected to western blotting, the probe pulled down less AGO in the tRF3008A-LNA-transfected cells than in the control cells. (Bottom). **E** qRT-PCR analysis of tRF3008A in the HA IP fractions, the pan-Ago IP fractions and Ago2 IP fractions from 293 T cells (left). qRT-PCR analysis of tRF3008A from the pan-Ago and control immunoprecipitation (IP) fractions from HCT116 cells and HT29 cells (Right). **F** HCT116 cells were co-transfected with tRF3008A mimetic and sh-AGO, and Western blotting analysis of AGO and FOXK1 expression was performed. RNAs isolated from these cells were subjected to real-time PCR. *: *P* < 0.05, ***: *P* < 0.001

To test if tRF-3 can enter RNA-binding complexes to perform sequenced-dependent microRNA-like functions, we performed sRAP-MS (small RNA Affinity Purification- Mass Spectrometry) by synthesized and biotin labeled tRF3008A mimetic to assess tRF3008A interacted protein. tRF-3008A was found to be highly associated with Argonaute proteins according to the potential binding scores (Fig. 5A & B) among a variety of potential partners (Fig. S5A & B). Gene Ontology (GO) indicated that RNA splicing and processing are shared functions of these proteins (Fig. 5C). Then, we further demonstrated the interaction between tRF3008A and AGO. A tRF-3008A probe was added lysate to HCT116 cells transfected with tRF3008A mimetic or scrambled tRF mimetic control. Western blot analysis showed no significant change in AGO protein expression from lysates prepared from these cells, but the tRF3008A probe was able to pull down AGO protein in the tRF3008A mimetic group (Fig. 5D, upper); however, in HT29 cells, AGO proteins were pulled down in the control group rather than in the tRF3008A-LNA group (Fig. 5D, down).

We purified Argonaute-associated RNAs from 293 T cells by using a monoclonal antibody with reactivity against all four human Argonaute proteins. Quantitative RT-PCR analysis of the coprecipitated RNA confirmed that tRF3008A was enriched in the pan-Ago immunoprecipitation (IP) fraction relative to a control IP (Fig. 5E). To dissect the binding affinity of tRF3008A for AGO2 and Pan-AGO, we performed IP of HA-tagged versions of human AGO2 and Pan-AGO that were transiently expressed in 293 T cells; the results demonstrate that tRF3008A is enriched in the IP fractions of each Argonaute protein but exhibited higher levels of expression in the pan-AGO fraction, suggesting that tRF3008A is specifically incorporated into silencing complexes containing each of the four Argonautes (Fig. 5E). Moreover, AGO silencing reversed tRF3008A-induced FOXK1 downregulation at both the protein and mRNA levels (Fig. 5F). Therefore, AGO is necessary for gene repression by tRF3008A.

### Low expression of serum and tissue tRF3008A correlates with advanced disease and metastasis in colorectal cancer

We next examined potential clinical significance of tRF3008A expression using a separate training cohort (*n* = 45) and validation cohort (*n* = 34). tRF3008A showed low expression in all CRCs and more selectively in larger tumors (*P =* 0.048). Higher tRF3008A expression was found in CRCs with better differentiation (*P* = 0.011). We additionally measured the tRF3008A expression in serum from CRC patients (*n* = 34), and found that lower tRF3008A expression is correlated with larger tumor size (*P* = 0.014) and more advanced clinical stage (Table 1).

**Fig. 6:**
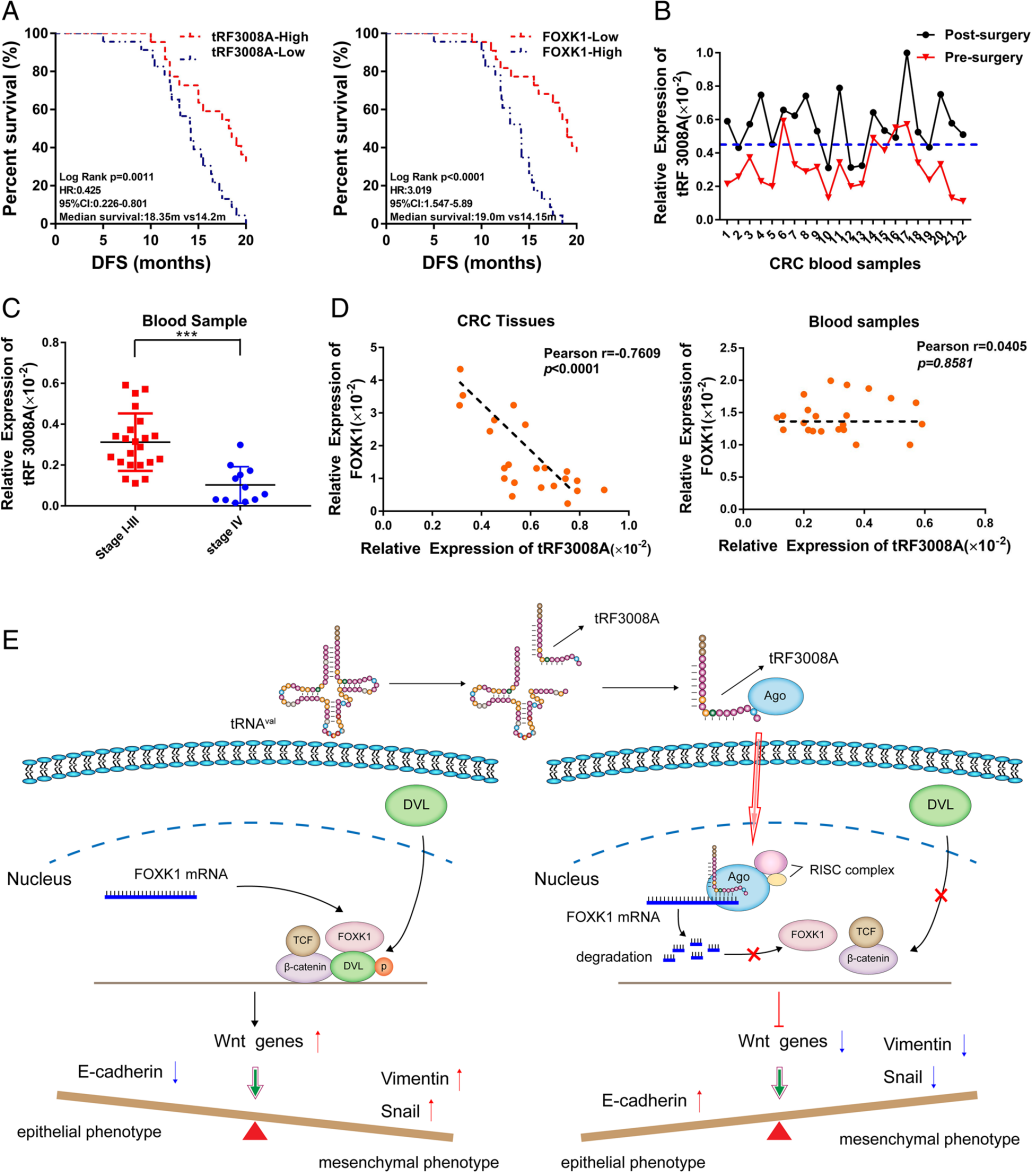
tRF3008A as a Novel Predictive Biomarker for colorectal cancer. **A** The expression of tRF3008A and FOXK1 was validated in a subset of 45 pairs of CRC and adjacent non-tumorous tissues. DFS analysis was performed by the Kaplan–Meier test and the log-rank method in the training cohort. **B** The expression of tRF3008A in plasma before and after surgery in 22 CRC patients was analyzed by qRT-PCR. **C**. RT-PCR was conducted to validate the expression of tRF3008A between CRC patients in clinical stages I-III (*n* = 22) and CRC patients in clinical stage IV (*n* = 12). **D** qPCR was performed to evaluate the expression correlation between tRF3008A and FOXK1 in CRC tissues and blood samples in the validation cohort (*n* = 22); Spearman’s rank correlation test was used. ***: *P* < 0.001. **E** Proposed working model. Schematic summarizing our proposed model for tRF3008A-mediated inhibition of the Wnt pathway in colorectal cancer cells. tRF-3008A binds to AGO proteins and induces subsequent destabilization of FOXK1 mRNA, which blocks the activation of Wnt signaling and EMT in colorectal cancer cells

To further investigate the potential impact of tRF3008A and FOXK1 on the prognosis of CRC patients, we analyzed their expression patterns in the clinical training cohort (*n* = 45). The tRF3008A high expression group showed a tendency to be associated with better DFS (*p* = 0.0011, HR = 0.425), while the FOXK1-high expression group showed poor DFS (*P<*0.001, HR =3.019) (Fig. 6A). Furthermore, multivariate Cox regression analysis revealed that high tRF3008A expression was an independent predictor for better prognosis in both clinical training cohorts (HR: 0.701, 95% CI: 0.458 to 1.005, *P* = 0.002, Table 2).

**Table 2:** Univariate and multivariate analysis for predictors of Disease-free survival in cohort1

Variable	Cohort1			
	Univariate survival analysis		Multivariate survival analysis	
	HR(95%CI)	*P*	HR(95%CI)	*P*
Age (≤ 51*vs* > 51)	1.548(0.848–2.825)	0.155		
Gender (female vs male)	1.184(0.638–2.199)	0.592		
Tumor size (> 5 cm vs ≤ 5 cm)	1.993(1.053–3.773)	0.034*	2.305(1.112–4.777)	0.025*
Tumor differentiation (Poor vs Well)	2.347(1.240–4.441)	0.009**	2.054(0.971–4.343)	0.060
Tumor invasion (T3 + T4 vs T1 + T2)	1.025(0.562–1.870)	0.935		
Tumor location (Proximal vs Distal)	2.696(1.262–5.759)	0.01*	3.064(1.411–6.654)	0.005**
ECGO (≥2 vs 0–1)	1.364(0.604–3.084)	0.455		
tRF3008A expression (Low vs High)	0.611(0.358–1.125)	0.002**	2.342(1.083–5.065)	0.031*

We also tested tRF3008A expression in patient blood samples (plasma) collected before and after surgery; interestingly, tRF3008A expression increased after surgery (Fig. 6B). RT-PCR was conducted to validate the expression of tRF3008A between CRC patients in clinical stagesI-III (*n* = 22) and CRC patients in clinical stage IV (*n* = 12), results showed that lower tRF3008A expression is correlated with more advanced clinical stage, suggesting that tRF3008A act as tumor suppressors in CRC (Fig. 6C). Additionally, qPCR was performed in CRC tissues and blood samples to confirm the correlation between tRF3008A expression and FOXK1 expression. We validated the negative correlation of tRF3008A and FOXK1 in CRC tissues, but not in the blood samples (Fig. 6D). Taken together, these findings support that reduced expression of tRF3008A might be a potential biomarker for poor prognosis in CRC patients.

## Discussion

An increasing amount of research supports the existence of highly abundant miRNA-like tRNA fragments in various cell types [15–18]. With new techniques deployed to sequence tRNA and tRFs, new tiRNAs and tRFs are discovered and used as useful non-invasive diagnostic biomarkers in colorectal cancer [19]. In this study, by employing small-RNA sequencing, we found a group of tRFs that were differentially expressed in colorectal cancer cells. Here, we identified 3-tRF that contains tumor-suppressive and metastasis-suppressive activity. We revealed that expression of the tRNA^val^-derived fragment tRF3008A repressed cell proliferation and cell progression in colorectal cancer via inhibition of the Wnt/β-catenin pathway in an AGO-dependent manner. It should be noted, however, that the effects of artificial high expression of a short RNA on pathways is inevitable as the induction of endogenous tRFs in cells is substantially more modest than exogenous transfection of synthetic tRF mimetics. Nonetheless, our rescue studies involving these fragments reveal robust in vitro effects resulting from their regulation in colorectal cancer. Moreover, our data demonstrate an additional explanation of how aberrant Wnt signaling influences colorectal cancer cells, even though the significance of the Wnt pathway in cancer progression has been documented for many cancer types. Interestingly, High levels of tRFs in CRC was reported to be due to the upregulation of tRNA demethylases ALKBH3, which indicated that tRNA derived tiRNA and tRFs might play different roles in biological regulation [19].

FOXK1 belongs to the family of forkhead box class K (FOXK) transcription factors with two critical domains: a forkhead-associated (FHA) domain and a winged helix (WH) DNA-binding domain. The FHA domain is required for the recognition of phosphopeptides and the WH DNA-binding domain is critical for transcriptional regulation [20, 21]. FOXK1 has been documented as a mediator in a wide spectrum of biological processes [22–24]. More importantly, it has been revealed that FOXK1 plays pivotal roles in tumor initiation and progression [25]. In colorectal carcinoma, FOXKs promote Wnt/β-catenin signaling by translocating DVL into the nucleus [14]. Here, in the reciprocal experiment, we demonstrate that FOXK1 leads to increased proliferative capacity of colorectal cells. These data further highlight the pro-proliferative effects of FOXK1 that support tumor growth. Interestingly, our findings reveal that FOXK1 mRNA is a predominant binding target for tRF3008A in colorectal cancer cells. We speculate that in CRC, tumor-suppressive tRFs impair the stability of FOXK1 mRNA and subsequently lead to the inhibition of Wnt/β-catenin signaling involving epithelial-to-mesenchymal transition (EMT).

This tRF-mediated post-transcriptional silencing is Argonautes proteins dependent. Argonautes proteins can be divided into the ubiquitous AGO clade and the germline-specific PIWI clade in mammals [11, 26]. There are four AGO clade proteins (AGO1–4) in humans expressed in specific cell types, which are loaded with microRNA [27]. Previous studies in recent decades have revealed that the Argonaute protein family acts as a key player in smRNA-directed transcriptional gene silencing (TGS) [28]. Binding to AGO proteins, they form miRNA-containing ribonucleoproteins (miRNPs) assembled at targeted mRNAs that trigger target mRNA degradation [29]. These small RNAs include miRNA, snoRNA and tiRNA [30–32]. Our findings expand the repertoire of endogenous smRNA-mediated TGS that have been previously described. As the molecular function and the primary localization of AGO proteins varies from different cell types and tissues, we didn’t differentiate between nuclear and cytoplasmic AGO proteins in this study, but further analysis of subcellular AGO protein localization will allow gauging of the extent and functional importance of AGO-dependent TGS. In contrast to tRFs-protein competitive replacement silencing reported by *Hani Goodarzi.*et al [33], our findings support a role for endogenous tRFs in destabilizing oncogenic transcripts through binding to AGO and in serving as guides in posttranscriptional silencing (Fig. 6E). These observations parallel those seen with other smRNA implicated in cancer progression—moderate post-transcriptional suppressive effects serve as regulatory effects in common oncogenic processes.

These functional tRFs are thought to be produced from ribonucleolytic processing of tRNAs by Dicer and RNase Z [6]. To better understand tRF biology and function, identification of the specific conditions that are important for tRF generation is essential. In parallel, many other oncogenes, including Ras, Raf, EGF receptor, and c-Myc, and oncoproteins, such as E6 and E7, are known to induce tRNA generation [15, 34]. Thus, it is an interesting hypothesis that these oncogenes may indirectly increase the levels of specific tRFs. Based on this evidence, we speculate two mechanisms that contribute to tRF3008A-mediated tumor suppression: the first is the induction of tumor-suppressive tRFs within specific tumor microenvironments, such as hypoxia and tumor mutation burden, and the second is related to upregulated AGO expression, as reported in colorectal cancer [35].

Tumor initiation and progression involve complicated genetic and epigenetic alterations. It was reported that 5′-tRF-GlyGCC in the plasma of CRC patients is dramatically increased in plasma of CRC patients compared to that of Health controls, suggesting that tRFs can be used as promising biomarkers for CRC diagnosis [19]. Interestingly, from the analysis of tRFs expression among CRC patients from different clinical stages, we found that highly metastatic colorectal cancer patients did not express a significant abundance of tRFs compared to that in patients in the early clinical stages, suggesting a potential suppressive role for these molecules in cancer progression. For the plasma tRFs, they can be produced by both tumor tissues and normal tissues so that plasma tRF3008A levels might not correlated with disease stages. After the removal of the tumor mass, the suppressive factors of tRFs production might be removed as well, that might be one of potential explanation of the increased after surgery. The amount of tRFs is also influenced by clearance, degradation and other physiological filtering events of the blood and lymphatic circulation. It remains unclear whether the observed changes in tRF abundance before and after surgery are the cause of the disease state or result from it. Therefore, future work will need to both investigate such differential tRF profiles and the context in which these tRFs are produced and released. In addition, considering limited samples for sequencing and tumor heterogeneity, we identified 16 differentially regulated tRFs or tiRNAs and only tRF3008A was uncovered. It would be difficult to obtain complete profile of differential expressed tRFs & tiRNAs from 3 pairs of sequenced tissues, but many other cancer-related tRFs or tiRNAs were found in CRCs [19, 36, 37], which suggested that the tRNA-derived small RNAs and its associated network could represent novel targets in colorectal cancer.

Taken together, our study investigated a 3-tRNA^Val^-derived fragment named tRF3008A that induces the destabilization of oncogenic transcripts through its direct binding to AGO. Further studies have demonstrated that tRF3008A can directly inhibit Wnt/β-catenin signaling by targeting FOXK1. These results could explain how aberrant Wnt/β-catenin signaling activation, which is driven by ncRNA fragments, is molecularly connected to cancer initiation and progression. As proof of principle for its ability to regulate endogenous target genes, tRF3008A was identified as a powerful master regulator that may be a novel target for reversing tumor progression. As we learn more about the function of ncRNAs and roles for nuclear AGO, it is exciting that we will better understand the potential for small RNAs to act as natural regulators of gene expression in the nucleus, which will in turn lead to recognition of novel intervention points for therapy.

## Conclusion

Our findings implicate specific tRNA-derived fragments as potential tumor-suppressive modulators of colorectal carcinogenesis, we identified a novel tRNA^Val^ -derived tRF3008A that decreases the stability of the oncogenic transcript FOXK1 in colon cancer cells by binding to AGO and serving as guides in post-transcriptional silencing. To the best of our knowledge, these data provide evidence that active tRFs can function as regulators and potential biomarkers and therapeutic targets in colorectal cancer.

## Supplementary Information

**Additional file 1.**

**Additional file 2: Figure S1.** Genome-wide profiling of tRFs in colorectal cancer. (A) Summary of the tRFs number which expressed in both of two groups and the tRFs number specifically expressed in one group. (B) RT-qPCR to validate the differential expression of top 3 differentially expressed tRFs in 10 pairs of CRC and adjacent non-tumorous tissues. (C) The expression level of tRNA^Val^ and tRF3008A in 7 CRC cell lines and human colon epithelial cell line using northern blot. (D) The expression level of tRNA^Val^ and tRF3008A in various groups using northern blot. Statistical significance is measured using a Student’s t test.**: *P* < 0.01, ***: *P* < 0.001. **Figure S2.** tRF3008A inhibits colorectal cancer growth and migration in vitro: HCT116 cells were transfected with scrambled tRF mimetic control or tRF3008A mimetic, and HT29 cells were transfected with scrambled LNA control or tRF3008A-LNA. (A) Line graph showing the quantification of relative Edu incorporation in different groups. (B) Active caspase-3 was analyzed by immunostaining (red). (C) qRT-PCR was performed to validate the expression of tRF3008A in subcutaneous tumors. (D&E) The TUNEL assay for apoptosis detection in subcutaneously implanted tumors with different tRF3008A expression levels. Data are presented as the means ± S.D. Statistical significance is measured using a Student’s t test. *: *P* < 0.05, **: *P* < 0.01, **: *P* < 0.01. **Figure S3.** (A&B). qPCR was performed to evaluate the expression correlation between tRF3008A and its potential targets (DDA1 and HOXC13) in CRC tissues. (*n* = 45); Spearman’s rank correlation test was used. **Figure S4.** tRF3008A inhibits the growth and EMT of CRC cells through the FOXK1/Wnt pathway. HCT116 were co-transfected with tRF3008A mimetic and sh-FOXK1 or co-treated with Wnt pathway activator (Wnt agonist 1); HT29 cells were co-transfected with tRF3008A-LNA and OE-FOXK1 or co-treated with Wnt pathway inhibitor (IWR-1-endo). (A) qPCR was performed to evaluate the related mRNA levels of Wnt/β-Catenin signaling molecules (C-Jun、c-Myc and CCND1). (B) Active caspase-3 was analyzed by immunostaining (red). (C & D) Cell migration and invasion abilities were evaluated by transwell migration and matrigel invasion assay. Data were represented as means ± SD from at least three independent experiments. *: *P* < 0.05, **: *P* < 0.01, ***: *P* < 0.001. **Figure S5.** tRF3008A modulates FOXK1 in an AGO-dependent manner. tRF3008A affinity purification Mass spectrometry assay was performed: (A). The ThermoFisher Scientific 1200 liquid phase system and Q Exactive mass spectrometer were used for the analysis and identification of the samples, the quality spectrum results showed that there was a mass spectrum peak. (B) STRINGdb protein-protein interaction analysis in tRF3008A related proteins.

**Additional file 3.**

## Data Availability

Not applicable.

## Abbreviations

CRC: Colorectal cancer
FBS: Fetal bovine serum
tRFs: tRNA-Derived Fragments
LNA: Locked nucleic acid
shRNA: Small interfering RNA
TUNEL: Terminal deoxynucleotidyl transferase (TdT) dUTP nick-endlabeling
DAPI: 4,6-diamidino-2-phenylindole
RIPA: Radioimmunoprecipitation assay
TBS: Tris-buffered saline
ANT: Adjacent non-tumorous tissue
RT-qPCR: Quantitative reverse transcription PCR
DFS: Disease-free Survival
EMT: Epithelial-mesenchymal transition
