# Supplementary material for: tRF3008A suppresses the progression and metastasis of colorectal cancer by destabilizing FOXK1 in an AGO-dependent manner
Source: J Exp Clin Cancer Res. 2022 Jan 22;41:32. doi: 10.1186/s13046-021-02190-4 (PMC8783529; doi:10.1186/s13046-021-02190-4)
Supplement: Supplementary file 1 — Additional file 1. [file 13046_2021_2190_MOESM1_ESM.doc]

**Supplementary Materials and Methods**

**Cell proliferation assay**

Cell viability was determined with a Cell Counting Kit-8(TransDetect® Cell Counting Kit). Generally, Cells (2.0×10^3^/well) were seeded into a 96-well plate subjected to various indicated treatments, 10μL CCK-8 was added to each well for 2h, and then the absorbance at 450 nm was measured. Each assay was performed in triplicate.

For the detection of DNA replication foci, Cell-Light TM EdU Kit (RiboBio, Cat. No. C10312) was applied according to the manufacturer’s instructions. Each assay was performed in triplicate.

**Apoptosis assay (Flow cytometry)**

The apoptosis was analyzed by ﬂow cytometry on a FACS Canto II ﬂow cytometer (BD Biosciences). Annexin V-FITC Apoptosis Detection Kit I (Cat.No.556547, BD Pharmingen, USA) was applied to assess apoptosis according to the manufacturer’s instructions. Brieﬂy, cells resuspended in 1x Binding Buffer at a concentration of 1 × 10^6^ cells/ml, were added to a tube containing both Annexin V-FITC reagent (5 µl) and a PI reagent (5 µl), incubating for 20 min at room temperature in the dark. Then the samples were analyzed by flow cytometry within 1 hour. The following controls are used to set up compensation and quadrants: 1). Unstained cells; 2). Cells stained with FITC Annexin V (no PI); 3). Cells stained with PI (no FITC Annexin V).

**Migration and invasion assays**

Cells were starved for 24 hr in 1640-based media supplemented with 0.2% FBS. Then cells were seeded into transwell upper chamber (Corning Inc costar®, USA). For migration assays, 5×10^4^ cells were plated in 0.8 µm inserts in 24-well plates with 100 µl of 1640 inside the insert and 600 µl of 10% 1640 outside the insert. For invasion assays, 5×10^4^ cells were seeded in the same conditions in Matrigel coated trans-well invasion chambers with a 0.8 mm pore size. Cells were incubated for 24 hr and then the inserts were washed twice with fresh PBS and fixed with methanol for 10 min. After fixation, non-migrating cells were carefully removed using a cotton swab. Fixed cells were stained with 0.1% crystal violet for 20 min. images were obtained using a computerized Olympus microscope.

**Real time PCR for non-small RNAs**

For non-small RNAs, total RNA was isolated using TRIzol reagent (Invitrogen). Total RNA (2 µg) was used for the synthesis of first-strand cDNA. qRT-PCR was performed using the SYBR Green Mix (TransStart® Top Green qPCR SuperMix). The reactions were performed with a 7500 Fast Real-Time PCR System. The relative expression of mRNA was normalized against β-Actin. The levels of mRNA were calculated using 2^−ΔCt^ or 2^−ΔΔCt^ method for relative quantification of expression, in which ΔCt = Ct (genes of interest)–Ct(β-actin), and ΔΔCt = ΔCt(case) -ΔCt(control).

**List of primers：**

tRF 3008A-F: F:5'- ATTCCGACGATCACCGGG-3’

tRF 3008A -R: F:5’- CGCTGCCGATCTTGGTGT-3’

tRF 1001 -F: 5’ -ATTCGCGACGATCGAAGCG-3'

tRF 1001-R: 5’ -CCGCCGTCCGATCTAAAATA-3’

tRF 3001 -F: 5’ - CAGTCCGACGATCATCCCAC -3'

tRF 3001-R: 5’ - TGCTCTTCCGATCTTGGTGG -3'

FOXK1-F: 5’-GGGAGTTCGAGTTCCTGATG-3’;

FOXK1-R:5’-AGAAGTGAGGCTCCTGGAAG-3’

ADAMTS4-F:5’-CAAGGTCCCATGTGCAACGT-3’

ADAMTS4-R:5’- CATCTGCCACCACCAGTGTCT-3’

DDA1-F: 5’-GCCCTCAGTCTACCTGCCTA-3’

DDA1-R: 5’-TCCTGGTCTCTCTTCTTGGC-3’

HOXC13-F: 5’-TGCAGCGGAGCGAGCCCC-3’

HOXC13-R:5’-TCAACAGGGATGAGCGCGTCGTG-3’

C-JUN-F: 5’-TCAGACAGTGCCCGAGATG-3’

C-JUN-R:5’- CTGCTGCGTTAGCATGAGTT-3’

c-Myc-F: 5’- GCTGCTTAGACGCTGGATTT-3’;

c-Myc-R:5’-CTCCTCCTCGTCGCAGTAGA-3’

CCND1-F: 5’- GACCTTCGTTGCCCTCTGT-3’

CCDD1-R:5’-TGAGGCGGTAGTAGGACAGG-3’

GAPDH-F:5’-ATGGGGAAGGTGAAGGTCG-3’;

GAPDH-R:5’-GGGGTCATTGATGGCAACAATA-3’

U6-F:5’-GCTTCGGCAGCACATATACTAAAAT-3’

U6-R:5’-CGCTTCACGAATTTGCGTGTCAT-3’

**Western blotting**

Whole-cell lysates were prepared using RIPA lysis buffer in the presence of protease inhibitors. Total cell lysates were separated using 10% SDS-PAGE, and transferred onto 0.2-μm PVDF membranes (Millipore, Bedford, MA) under a constant 300-mA for 90min and then incubated with the primary antibodies at 4°C overnight. Next day ,after incubating with an HRP-conjugated secondary antibody for 1 h at 37°C, the bands were automatically visualized using the ChemiDoc XRS+ System (Bio-Rad, Hercules, CA) and quantitatively analyzed with Image Lab software (Bio-Rad). β-Actin protein expression was used as the internal control.

**List of Antibody：**

FOXK1: ab85999, ABCAM, WB (1:2000)

C-JUN: 9165T, CST, WB (1：1000)

CCND1: 2978T, CST, WB (1：1000)

Vimentin: ab8069, ABCAM, WB (1:1000)

Snail: 3879T, CST, WB (1：1000)

E-cadherin: ab40772, ABCAM, WB (1:10000)

Cleaved caspase-3: 9664, CST, WB (1：1000), IHC（1：2000）, IF (1:400)

Ki67：ab16667，ABCAM，IHC (1:200)

MMP-9: 13667, CST, IHC (1:325)

Pan-AGO: MABE56，Millipore, WB (1:500)

AGO2: ab233727, ABCAM

**Northern blotting**

Northern blotting was performed by Northern Max™ Kit (Thermo fisher, AM1940) according to the User guide. Briefly, total RNA (30-50 µg) was separated by on 18% denaturing polyacrylamide gels, then the separated gel was transferred onto the nylon Membranes (Thermo fisher, AM10100). The membrane was then UV-crosslinked and

hybridized with bio-labeled DNA probes.

**List of Northern blot probe sequences**

U6 (5'-3'): GTGCTCGCTTCGGCAGCACATATACTAAAATTGGAACGATACAGAGAAGATTAGCATGGCCCCTGCGCAAGGATGACACGCAAATTCGTGAAGCGTTCCATATTTT；

tRF3008A (5'-3'): ACA TGG TGT TTC CGC CCG GT

**Immunoﬂuorescence (IF) staining**

Cells were cultured in chambered culture slides overnight at 37° C, fixed in 4% formaldehyde for 10 min at room temperature, then permeabilized for 15 min in 0.5% Triton X-100, followed by blocking with 3% bovine serum albumin for 1 h. And then the primary antibody was applied to the slides overnight at 4°C, and subsequently incubated with the corresponding Alexa Fluor 594-conjugated secondary antibodies. Images were captured using a TCS SP8 confocal microscope (Leica, Wetzlar, Germany).

**Hematoxylin and eosin (H&E) staining and** **Immunohistochemistry (IHC):**

Xenograft tumors were fixed in 10% formalin, dehydrated, and embedded in paraffin and cut into 5-μm sections. Then sections were stained with hematoxylin and eosin (H&E). For Immunohistochemistry, after undergoing dewaxing and hydration, antigen retrieval, and blocking of endogenous peroxidase activity, the slides were then incubated with primary antibodies at 4°C overnight. The number of macroscopic nodules were then recorded for each section. An unpaired t test was used to test for significant variations.

**Reagent（Inhibitor and Activator）**

Wnt pathway inhibitor IWR-1-endo(S7086) and Wnt pathway agonist 1(S8178) were purchased from Selleck (USA)

**Plasmid construction and viral infection**

The human FOXK1 overexpression plasmid and siRNA (AGO1, AGO3 and FOXK1) was synthesized (Ribobio, Guangzhou, China). Briefly, cells (2×10^5^/well) were plated into 6-well plates and incubated overnight. siRNA or Control siRNA were diluted in 250μl Opti-MEM (Gibco) and transfected with the concentration of 100 nmol. Then, Lipofectamine 3000 transfection reagent were used. The siRNA sequences were depicted below: FOXK1 5’-CCAUCAAGAUCCAGUUCAC- 3’, Ago, 5’-GAGAAGAGGUGCUCAAGAAUU-3’
