# Supplementary material for: tRF3008A suppresses the progression and metastasis of colorectal cancer by destabilizing FOXK1 in an AGO-dependent manner
Source: J Exp Clin Cancer Res. 2022 Jan 22;41:32. doi: 10.1186/s13046-021-02190-4 (PMC8783529; doi:10.1186/s13046-021-02190-4)
Supplement: Supplementary file 2 — Additional file 2: Figure S1. Genome-wide profiling of tRFs in colorectal cancer. (A) Summary of the tRFs number which expressed in both of two groups and the tRFs number specifically expressed in one group. (B) RT-qPCR to validate the differential expression of top 3 differentially expressed tRFs in 10 pairs of CRC and adjacent non-tumorous tissues. (C) The expression level of tRNAVal and tRF3008A in 7 CRC cell lines and human colon epithelial cell line using northern blot. (D) The expression level of tRNAVal and tRF3008A in various groups using northern blot. Statistical significance is measured using a Student’s t test.**: P < 0.01, ***: P < 0.001. Figure S2. tRF3008A inhibits colorectal cancer growth and migration in vitro: HCT116 cells were transfected with scrambled tRF mimetic control or tRF3008A mimetic, and HT29 cells were transfected with scrambled LNA control or tRF3008A-LNA. (A) Line graph showing the quantification of relative Edu incorporation in different groups. (B) Active caspase-3 was analyzed by immunostaining (red). (C) qRT-PCR was performed to validate the expression of tRF3008A in subcutaneous tumors. (D&E) The TUNEL assay for apoptosis detection in subcutaneously implanted tumors with different tRF3008A expression levels. Data are presented as the means ± S.D. Statistical significance is measured using a Student’s t test. *: P < 0.05, **: P < 0.01, **: P < 0.01. Figure S3. (A&B). qPCR was performed to evaluate the expression correlation between tRF3008A and its potential targets (DDA1 and HOXC13) in CRC tissues. (n = 45); Spearman’s rank correlation test was used. Figure S4. tRF3008A inhibits the growth and EMT of CRC cells through the FOXK1/Wnt pathway. HCT116 were co-transfected with tRF3008A mimetic and sh-FOXK1 or co-treated with Wnt pathway activator (Wnt agonist 1); HT29 cells were co-transfected with tRF3008A-LNA and OE-FOXK1 or co-treated with Wnt pathway inhibitor (IWR-1-endo). (A) qPCR was performed to evaluate the related mRNA level [file 13046_2021_2190_MOESM2_ESM.docx]

**Supplementary Figures**

**
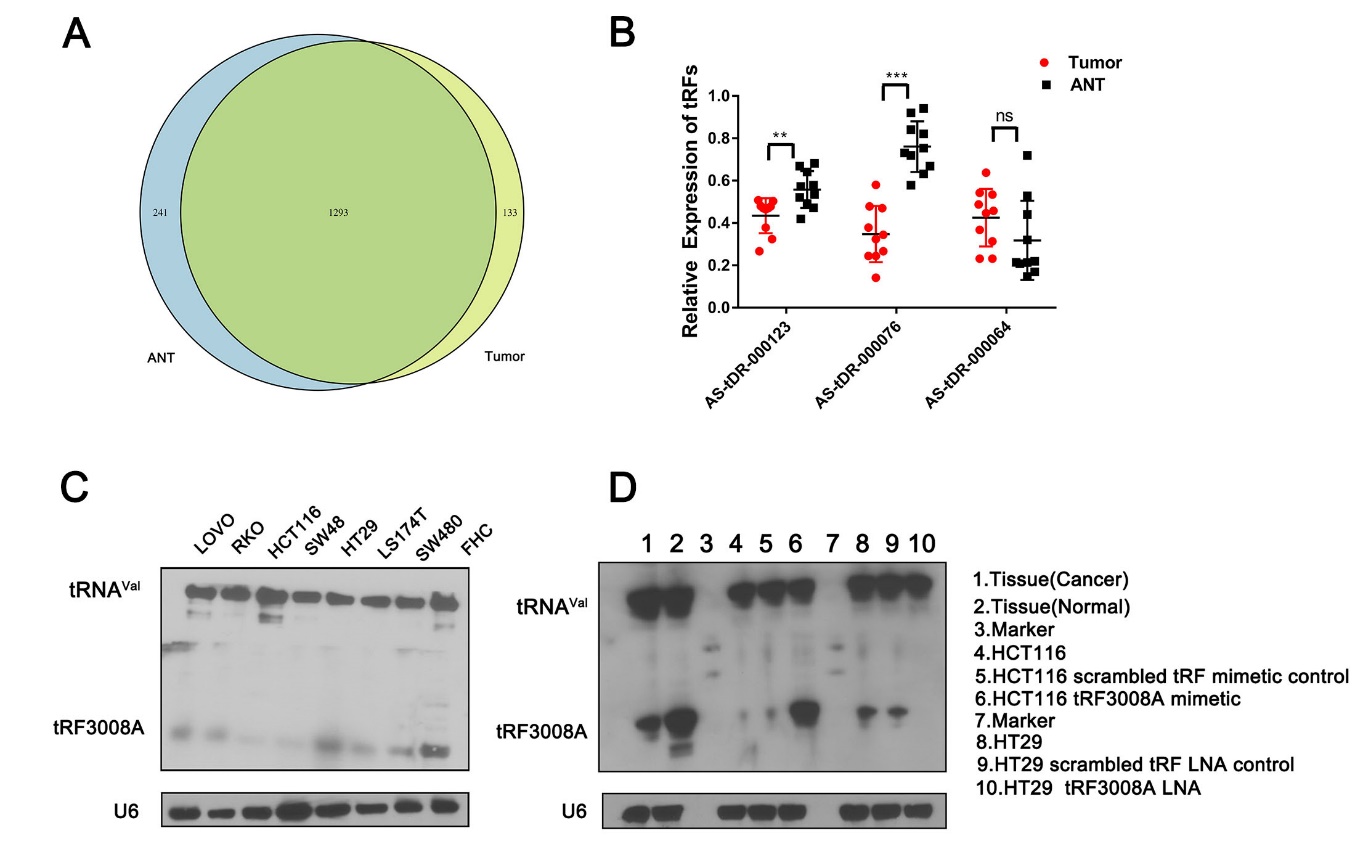
**

**Figure S1:** Genome-wide profiling of tRFs in colorectal cancer. (A) Summary of the tRFs number which expressed in both of two groups and the tRFs number specifically expressed in one group. (B) RT-qPCR to validate the differential expression of top 3 differentially expressed tRFs in 10 pairs of CRC and adjacent non-tumorous tissues. (C) The expression level of tRNA^Val^ and tRF3008A in 7 CRC cell lines and human colon epithelial cell line using northern blot. (D) The expression level of tRNA^Val^ and tRF3008A in various groups using northern blot. Statistical significance is measured using a Student’s t test.**: P < 0.01, ***: P < 0.001.

**
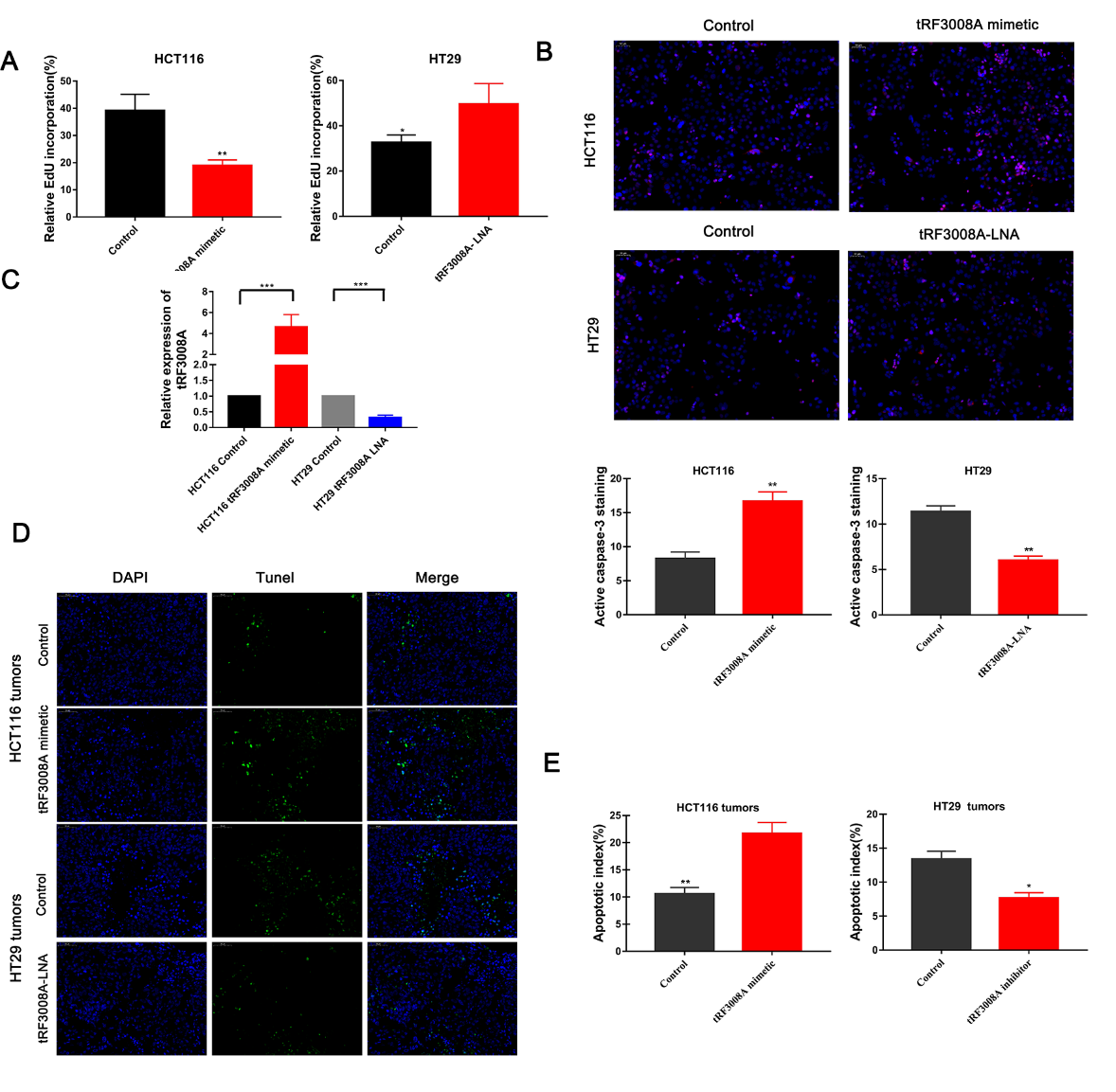
**

**Figure S2:** tRF3008A inhibits colorectal cancer growth and migration in vitro: HCT116 cells were transfected with scrambled tRF mimetic control or tRF3008A mimetic, and HT29 cells were transfected with scrambled LNA control or tRF3008A-LNA. (A) Line graph showing the quantification of relative Edu incorporation in different groups. (B) Active caspase-3 was analyzed by immunostaining (red). (C) qRT-PCR was performed to validate the expression of tRF3008A in subcutaneous tumors. (D&E) The TUNEL assay for apoptosis detection in subcutaneously implanted tumors with different tRF3008A expression levels. Data are presented as the means ± S.D. Statistical significance is measured using a Student’s t test. *: *P* < 0.05, **: *P* < 0.01, **: *P* < 0.01.

**
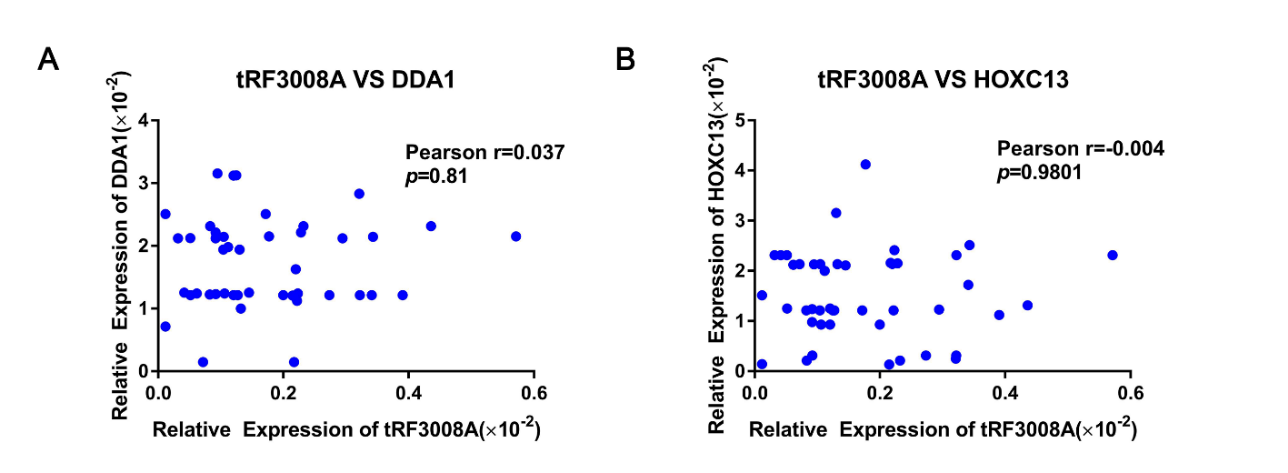
**

**Figure S3** (A&B). qPCR was performed to evaluate the expression correlation between tRF3008A and its potential targets (DDA1 and HOXC13) in CRC tissues. (n = 45); Spearman’s rank correlation test was used.


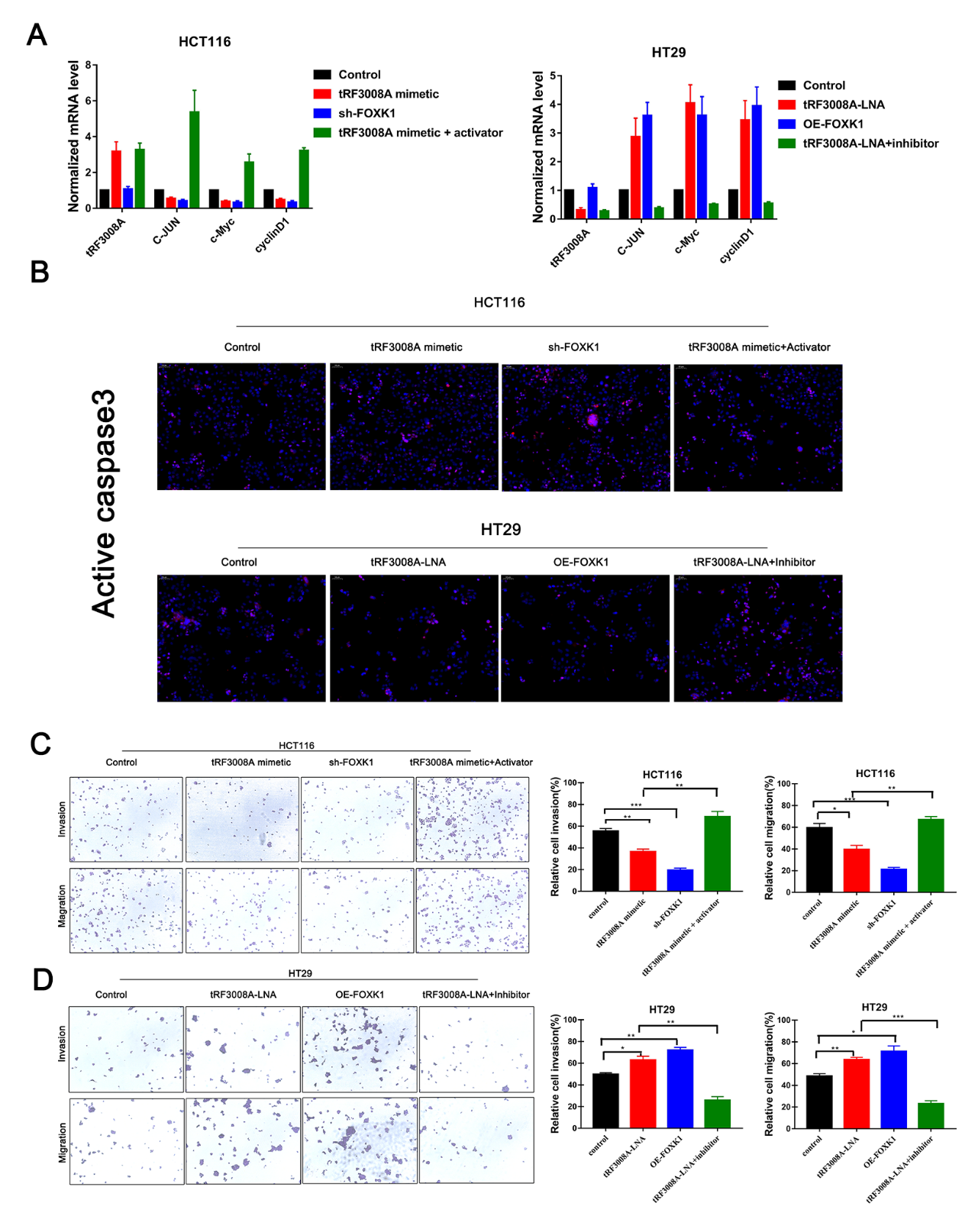


**Figure S4:** tRF3008A inhibits the growth and EMT of CRC cells through the FOXK1/Wnt pathway. HCT116 were co-transfected with tRF3008A mimetic and sh-FOXK1 or co-treated with Wnt pathway activator (Wnt agonist 1); HT29 cells were co-transfected with tRF3008A-LNA and OE-FOXK1 or co-treated with Wnt pathway inhibitor (IWR-1-endo). (A) qPCR was performed to evaluate the related mRNA levels of Wnt/β-Catenin signaling molecules (C-Jun、c-Myc and CCND1). (B) Active caspase-3 was analyzed by immunostaining (red). (C & D) Cell migration and invasion abilities were evaluated by transwell migration and matrigel invasion assay. Data were represented as means ± SD from at least three independent experiments. *: *P* < 0.05, **: *P* < 0.01, ***: *P* < 0.001.


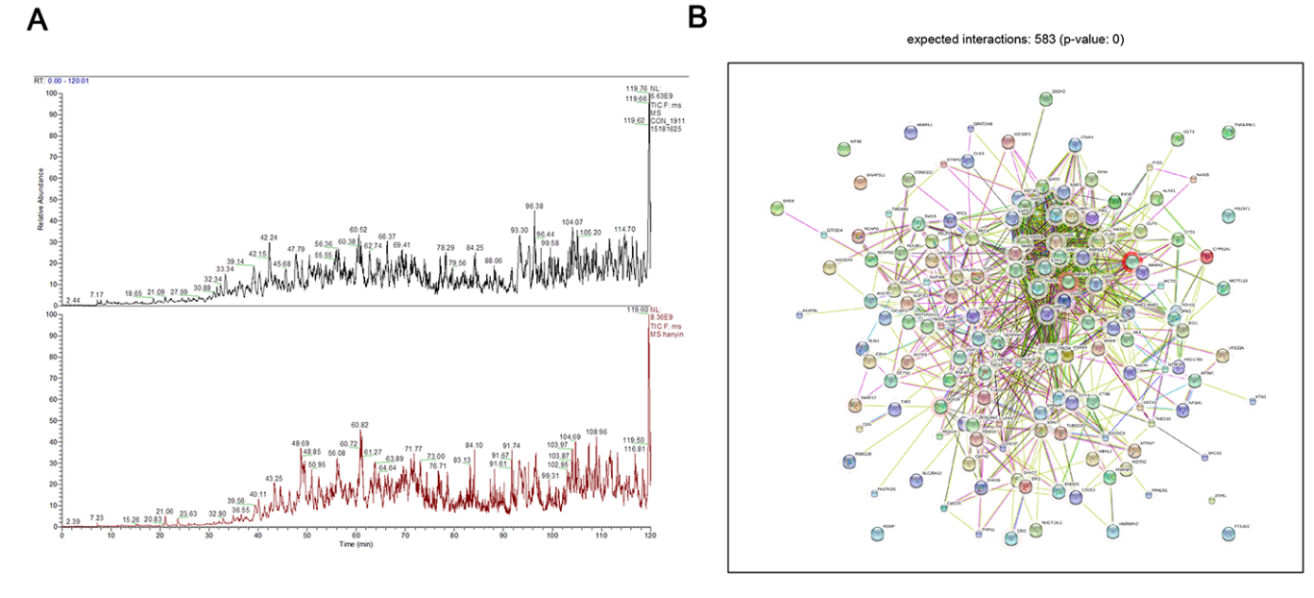


**Figure S5:** tRF3008A modulates FOXK1 in an AGO-dependent manner. tRF3008A affinity purification Mass spectrometry assay was performed: (A). The ThermoFisher Scientific 1200 liquid phase system and Q Exactive mass spectrometer were used for the analysis and identification of the samples, the quality spectrum results showed that there was a mass spectrum peak. (B) STRINGdb protein-protein interaction analysis in tRF3008A related proteins.
